# Perioperative hemostatic management of patients with type A aortic dissection

**DOI:** 10.3389/fcvm.2023.1294505

**Published:** 2023-11-20

**Authors:** Gabor Erdoes, Aamer Ahmed, Stephan D. Kurz, Daniel Gerber, Daniel Bolliger

**Affiliations:** ^1^ Department of Anesthesiology and Pain Medicine, Inselspital, University Hospital Bern, University of Bern, Bern, Switzerland; ^2^ Consultant Cardiothoracic Anaesthesiologist, Department of Anaesthesia and Critical Care, Glenfield Hospital, University Hospitals of Leicester NHS Trust, Leicester, United Kingdom; ^3^ Department of Cardiothoracic and Vascular Surgery, Deutsches Herzzentrum der Charité (DHZC), Berlin, Germany; ^4^ Clinic for Anaesthesia, Intermediate Care, Prehospital Emergency Medicine and Pain Therapy, University Hospital Basel, University of Basel, Basel, Switzerland

**Keywords:** type A aortic dissection, cardiac surgery, cardiopulmonary bypass, coagulopathy, coagulation monitoring, viscoelastic testing, blood products, transfusion

## Abstract

Coagulopathy is common in patients undergoing thoracic aortic repair for Stanford type A aortic dissection. Non-critical administration of blood products may adversely affect the outcome. It is therefore important to be familiar with the pathologic conditions that lead to coagulopathy in complex cardiac surgery. Adequate care of these patients includes the collection of the medical history regarding the use of antithrombotic and anticoagulant drugs, and a sophisticated diagnosis of the coagulopathy with viscoelastic testing and subsequently adapted coagulation therapy with labile and stable blood products. In addition to the above-mentioned measures, intraoperative blood conservation measures as well as good interdisciplinary coordination and communication contribute to a successful hemostatic management strategy.

## 1. Introduction

Bleeding is a common and severe complication in patients suffering from acute Stanford type A aortic dissection (ATAAD), often requiring extensive administration of blood products and coagulation factor concentrates. To a relevant extent, bleeding is associated and due to the high incidence of perioperative coagulation disorders. In coagulopathic ATAAD patients, a hospital mortality rate of up to 20% has been reported (1).

Coagulopathy in ATAAD patients is commonly complex and multifactorial. Important contributors include hypothermia, hyperfibrinolysis, extensive hemodilution, administration of high doses of unfractionated heparin, preoperative antiplatelet and antithrombotic therapy and pre-existing coagulopathy. Patients suffering from ATAAD often have cardiovascular risk factors and, therefore, are regularly pre-treated with anticoagulants including vitamin K antagonists (VKA), direct oral anticoagulants (DOACs) and/or antiplatelet agents. Because of the urgent care nature of ATAAD, recent intake (and thus relevant plasma drug levels) of such agents is common. Guidelines from different societies of cardiovascular anesthesia (2–4) regarding the perioperative intake of anticoagulants and antiplatelets often are not applicable for emergent surgery. In addition to acquired coagulopathy due to antiplatelets and anticoagulants or inherited coagulation disorders, a specific coagulopathy in ATAAD patients has been suggested.

The aim of this narrative review is to describe the coagulopathic challenges in patients undergoing emergent cardiac surgery due to type-A aortic dissection and give an overview in potential treatment options.

## 2. Coagulopathy associated with type A aortic dissection

### 2.1. Pathomechanism

**Figure 1:**
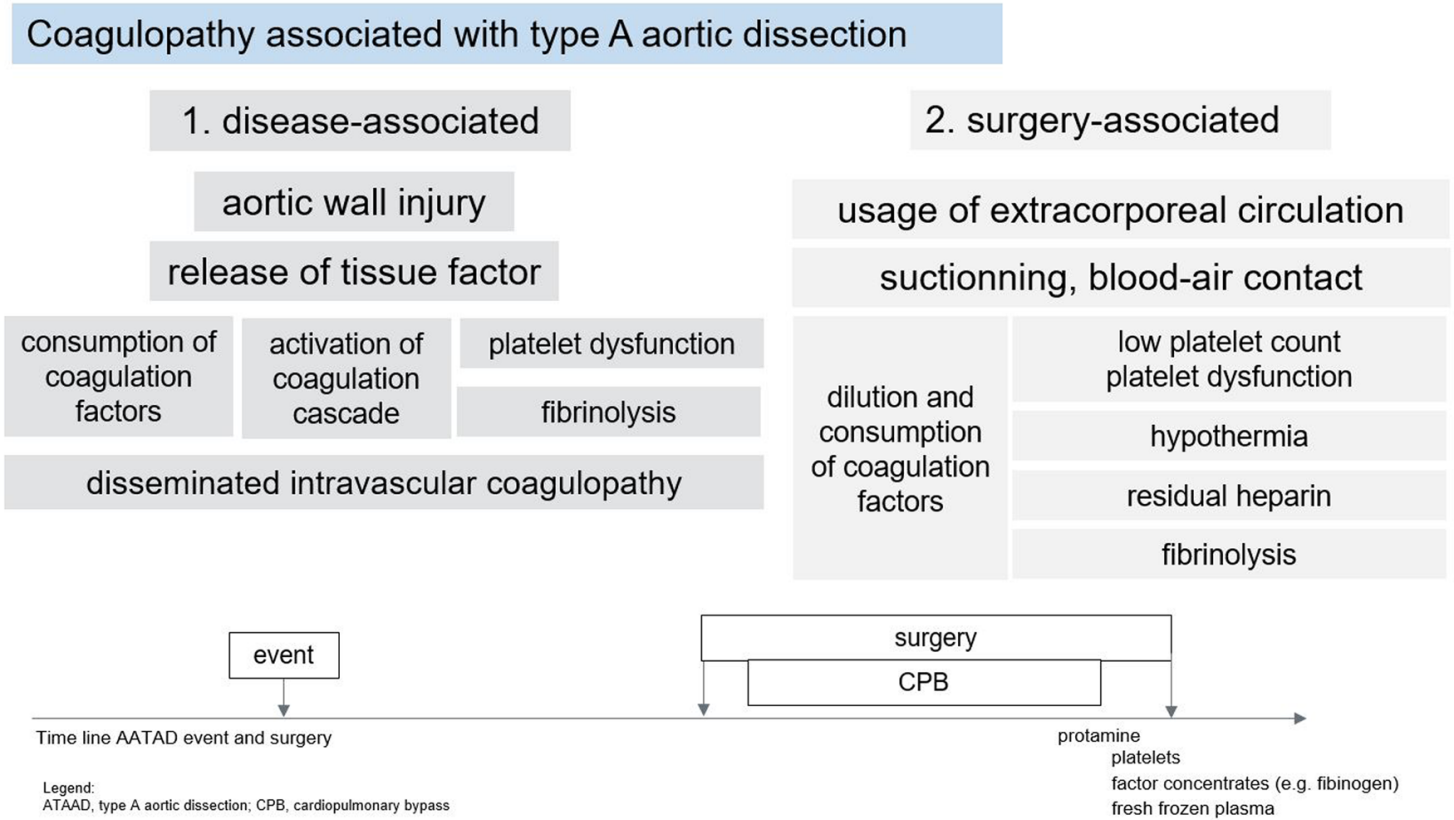
Coagulopathy associated with type A dissection.

After an aortic wall injury in ATAAD, the blood enters the false aortic lumen, which is not covered by endothelium. Thereby, blood comes in contact with the subcutaneous collagen and the aortic middle smooth muscular cells leading to massive secretion of tissue factor (TF) into circulation. TF release might eventually results in extensive coagulation activation. Such coagulation activation finally might end up with hyperfibrinolysis (Figure 1).

Acute coagulopathy of ATAAD is not well defined but it has been associated with a specific coagulation disorder that shares some properties with disseminated intravascular coagulopathy (DIC) and consumption of coagulation factors. Classical DIC is characterized by systemic activation of different coagulation pathways, which leads to concomitant thrombotic and bleeding tendency. In addition, enhanced fibrinolysis is commonly present in DIC because the endogenous fibrinolytic system is activated in parallel with increased coagulation to regulate and limit clot formation. In specific clinical conditions, such as massive trauma or post-partum hemorrhage, (hyper-) fibrinolysis dominates over prothrombotic complications and results in a bleeding tendency. Further, both massive systemic coagulation activation and extensive fibrinolysis eventually result in a relevant consumption of clotting factors, especially platelets and fibrinogen (5).

Finally, a former study using laser light-scattering methods has suggested that platelet aggregation is suppressed in patients with ATAAD, leading to general platelet dysfunction with acute aortic dissection and smaller platelet clots (6). The clinical relevance of this *in vitro* finding is unclear.

### 2.2. Laboratory coagulation testing

There is no gold standard laboratory diagnostic testing for DIC with enhanced fibrinolysis and consumption. Experimental laboratory testing might involve determination of D-dimers (or fibrin and fibrinogen degradation products; FDP), plasmin-alpha 2-plasmin inhibitor, and thrombin-antithrombin complex (TAT). In most clinical settings, laboratory testing of hyperfibrinolysis might be limited to determination of D-dimer and findings secondary to hyperfibrinolysis including hypofibrinogenemia and thrombocytopenia.

Coagulation changes with ATAAD usually results in evidence of increased thrombin formation, fibrinolytic activity and consumption of coagulation factor with high D-dimer levels and significantly increased levels of TAT, F1+2 (7, 8), and FDPs, whereas fibrinogen levels and platelet count are usually low or at least decreased (8, 9). Accordingly, D-dimer levels are usually higher and fibrinogen levels and platelet count are lower than in patients undergoing elective aortic procedures (1). Of note, levels of D-dimers are usually higher in patients with ATAAD than in patients with intramural hematoma or in patients with type-B aortic dissection (1, 5, 9, 10). In agreement, in viscoelastic testing including thromboelastometry, thromboelastography or techniques based on sonic estimation of elasticity via resonance (SEER), characteristic changes consistent with activation of the coagulation system and consumption of clotting factors have been described (5). Low fibrinogen levels and thrombocytopenia typically results with low amplitudes in both the FIBTEM and EXTEM test when using thromboelastometry (5). However, according to the authors' experiences, ATAAD patients often present with normal findings in the preoperative viscoelastic testing.

### 2.3. Anatomical and time considerations

It has been suggested that D-dimer levels may be related to the size of the contact area between thrombosis and blood of patients with ATAAD. With other words, the larger the extension of aortic dissection, the higher the activation of coagulation, consumption and hyperfibrinolysis. In agreement, in two recent cohort studies including nearly 350 patients with ATAAD, the increased DIC scores correlated with the larger diameter and length of the false lumen, with the greater thickness of the dissection membrane, and with the communicating-type ATAAD (11, 12).

Further, the longer symptom duration might be associated with the more severe coagulopathy and higher bleeding risk after surgery (13). The time between first symptoms to surgery might be associated with different levels of coagulation activation, potentially due to ongoing dissection of the aortic wall. The latter has to be taken into account when comparing different studies and supports emergent surgical therapy of ATAAD. In accordance with such assumptions, a recently published cohort study found that the symptom duration was an independent predictor of massive bleeding (OR: 0.974 per our increment, 95% CI, 0.950–0.999; *P *= 0.041) (1).

### 2.4. Coagulopathy associated with surgery requiring hypothermic circulatory arrest

Surgical repair of ATAAD is often complex and commonly requires the use of hypothermic circulatory arrest. Whereas the optimal temperature for cerebral (organ) protection is not well defined, many institutions nowadays apply moderate rather than deep hypothermia (14). However, thrombin generation, fibrin synthesis and fibrinolysis can be adversely be affected by hypothermia and acidosis (15). Establishing “coagulation-friendly” conditions including normal pH and normothermia is a pre-exquisite for successful coagulation management in bleeding patients after ATAAD repair.

Hemodilution might differently affect thrombin generation and fibrin formation. Reduced thrombin activation due to low factor levels might be partially compensated by lower activity of endogenous antithrombin and other protease inhibitors, whereas plasma fibrinogen rapidly decreased proportional to the extent of hemodilution (15). Development of platelet count is difficult to predict due to potential mobilization from bone marrow. To adequately and timely evaluate coagulation status and treat coagulopathic bleeding, recent European and American guidelines and experts' opinion recommend the administration of coagulation factors guided by viscoelastic testing (3, 4, 16).

## 3. Preoperative patient assessment

### 3.1. Assessment of preoperative therapy

Antithrombotic drugs are frequently used to prevent or treat various common cardiovascular disorders including coronary heart disease, peripheral vascular atherosclerosis, and atrial fibrillation (2). Two main classes of oral antithrombotic drugs are on the market, antiplatelets and anticoagulants. Aspirin and P2Y_12_ inhibitors, either alone or as dual antiplatelet therapy (DAPT) are typically prescribed antiplatelets. Among oral anticoagulants, VKAs and DOACs are commonly used (17, 18).

The number of patients chronically managed with such drugs might be increasing, and relatively common among ATAAD patients due to common risk factors and comorbidities. Whereas in the elective setting, recent guidelines suggests typical stopping intervals (3, 4), the evidence for their optimal perioperative treatment in the urgent and emergent setting remains limited (2).

### 3.2. Preoperative laboratory coagulation testing

The coagulation of patients with emergent surgery is clinically difficult to assess. Of note, the evaluation of the last intake of the antiplatelets and anticoagulant drugs and the assessment of the drug's activity in the patient seems eminent. The conventional laboratory coagulation testing including prothrombin time (PT), international normalized ratio (INR), activated prothrombin time (aPTT), fibrinogen levels, and platelet count seems justified as a minimum in each patient. Whether additional coagulation testing including D-dimer, FDP or specific coagulation factor levels might be beneficial for the patient's treatment and outcome is unclear. Coagulation testing seems to be especially important in patients with recent intake of anticoagulant drug. For VKA, testing of the international normalized ratio (INR) might be most adequate. For DOACs, the global coagulation tests including prothrombin time and activated partial thromboplastin time with values beyond normal range suggests elevated DOAC levels. However, normal ranges in conventional coagulation tests are not suited to rule out DOAC concentrations above a threshold of 30 ng/ml (19), a level that is thought not to affect coagulation (20). Instead, the specific coagulation testing should be applied. In patients with factor (F) Xa inhibitors such as rivaroxaban, apixaban or edoxaban, the use of drug-specific calibrated anti-Xa tests is recommended (19). If not available, the chromogenic FXa assays calibrated for unfractionated heparin, which is available in many institutions, can reliably estimate the anti-Xa activity of FXa inhibitors (21–23). Strong to very strong correlations between FXa inhibitor levels and heparin-calibrated anti-FXa assays over a broad range of drug concentrations (20–500 ng/ml) have been shown (21, 22). In patients on dabigatran, the use of the diluted thrombin time (dTT) is suggested to optimally estimated dabigatran levels. Further, a thrombin time (TT) within normal range excludes relevant activity of dabigatran (24).

**Figure 2:**
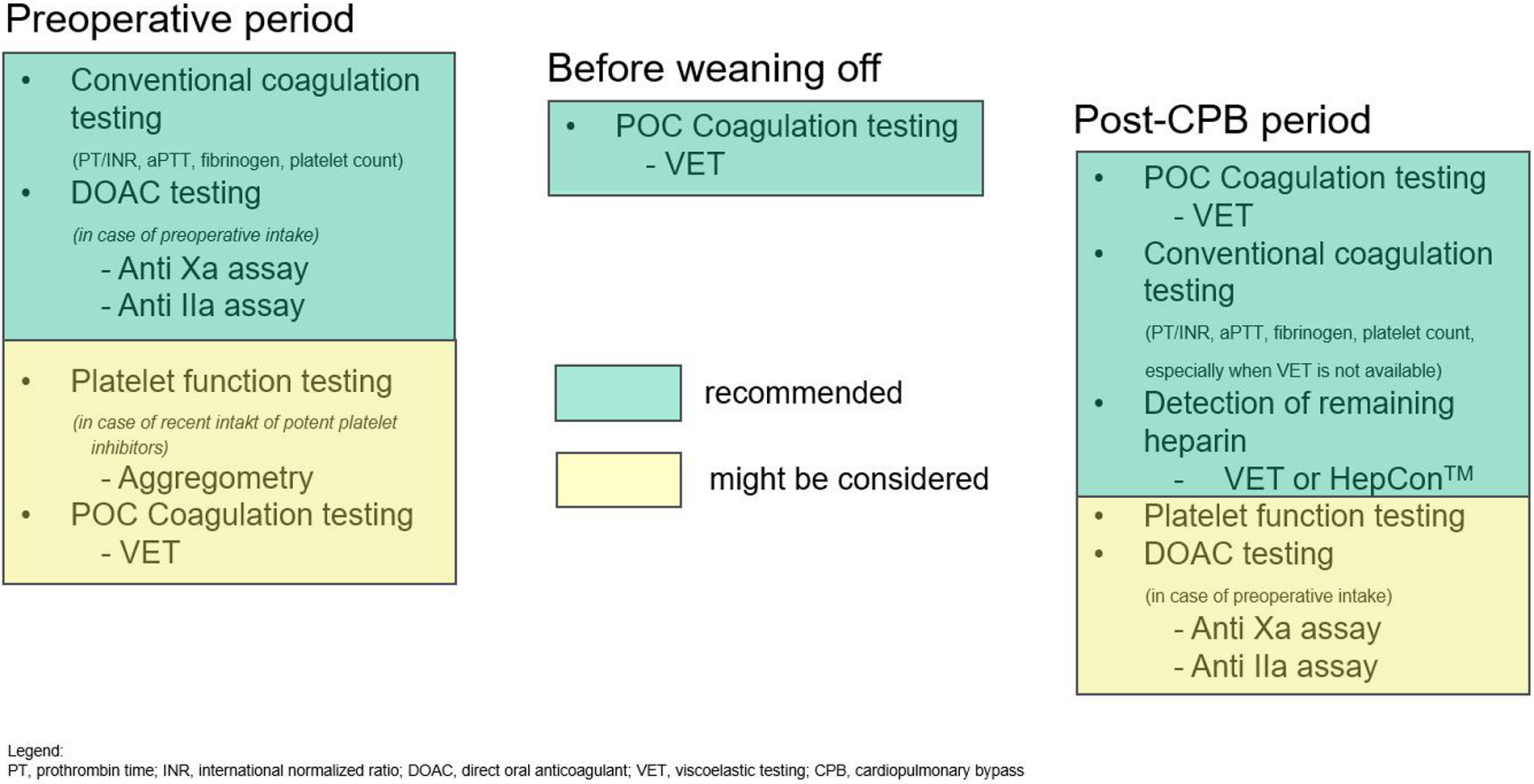
Suggested sequence of coagulation testing in the perioperative course.

In respect to patients with recent intake of platelet inhibitors, most anesthesiologists and cardiac surgeons are comfortable with managing emergent patients for ATAAD surgery despite recent aspirin intake (18). However, the recent administration of potent P2Y_12_ receptor inhibitors including clopidogrel, prasugrel or ticagrelor remains a major challenge in patients with emergent major surgery (18). Guidelines recommend stopping such potent platelet inhibitors 5–7 days before surgery to reduce perioperative bleeding risk (3, 4). Such a strategy is commonly not feasible in patients with ATAAD due to the emergency nature of the procedure. The use of different point-of-care (POC) platelet function analyzers has suggested solving this dilemma. However, the predictive values for postoperative hemorrhage and transfusion requirements is low, and there is a relevant variability between and within POC platelet function analyzers available for clinical use (18). Although such devices might be helpful in special patients and cases, a general and liberal use of platelet function testing is not recommended (3). Finally, it is unclear at the moment whether the preoperative viscoelastic testing including thromboelastometry, thromboelastography or SEER-based techniques will have additional value to conventional testing before emergent surgery in ATAAD patients (Figure 2).

### 3.3. Predictive value of preoperative coagulation markers

Laboratory evidence of hyperfibrinolysis in ATAAD patients (e.g., high D-dimer, low fibrinogen, low platelet count) was associated with worse outcome and increased mortality in a prospective cohort study including 206 patients (8). Of note, time form diagnosis to surgery was below 24 h in this study (8).

In agreement, the concentration of fibrinogen has been shown to be significantly lower before surgery in ATAAD patients as compared to patients undergoing similar elective aortic surgery in several studies (1, 5, 9). Low fibrinogen levels at hospital admission in ATAAD patients has been shown to be an independent predictor of in-hospital mortality (5). Further, it has been shown that the degree of platelet consumption in ATAAD patients and hence the lower platelet count before surgery was associated with worse outcome (25). This study included 183 consecutive patients with confirmed ATAAD admitted to a Chinese academic hospital. Patients were divided into quintiles according to their admission platelet count. In-hospital mortality was 39% in patients in quintile 1 (platelet count ≤119 × 10^9^/L), 8%–11% in quintile 2 to 4 (platelet count 120–228 × 10^9^/L), and only 3% in patients from quintile 5 (platelet count >228 × 10^9^/L) (25).

Taken to together, both low fibrinogen levels and low platelet count at hospital admission seem to be associated with worse outcome. These laboratory findings might be explained by fibrinogen and platelet depletion due to activation, consumption and hyperfibrinolysis (9, 26). Therefore, low platelet count and low fibrinogen levels might be rather a marker of more severe dissection than a direct cause for mortality. However, it is unclear at the moment whether the more severe coagulopathy due to more extended dissection eventually leads to worse outcome.

The predictive value of viscoelastic testing in emergent ATAAD patients is not well defined and clinical data are scarce. However, viscoelastic coagulation testing strongly depends on platelet count and fibrinogen level (27), both of them with predictive value on outcome. In addition, viscoelastic testing is rather sensitive for systemic hyperfibrinolysis (27). At the moment, no firm conclusion can be drawn about their predictive value on the benefit of viscoelastic coagulation testing in the preoperative setting of ATAAD patients.

## 4. Preoperative therapeutic interventions with evidence of coagulopathy

### 4.1. Pharmacological interventions

There are no specific interventions shown to improve acute coagulopathy associated due to ATAAD and/or to improve outcome of these patients. It is suggested that emergent surgery within short time after admission might limit ATAAD specific coagulopathy. The general and liberate use of tranexamic acid (TXA), administration of fibrinogen concentrate or transfusion of platelet concentrates in patients with evidence of low platelet count, low fibrinogen levels, or high D-dimers in the preoperative setting seems not recommendable. Such therapies might be considered as a last ditch therapy in patients otherwise not suitable for surgical interventions (28).

Further, it is unclear whether and how ATAAD patients with acquired coagulopathy due to intake of platelet inhibitors and anticoagulants should be treated before emergent surgery. The authors of this article suggest no specific therapy before surgery in such patients. In patients with preoperative treatment with P2Y_12_ receptor inhibitors, the transfusion with platelet concentrates rather after than before cardiopulmonary bypass (CPB) might be suggested. Some evidence suggests that in patients treated with ticagrelor, the use of a cytokine hemoabsorber (CytoSorb®; Cytosorbents, Monmouth, Junction, NJ) might be helpful. Two retrospective analysis in patients undergoing cardiac surgery with high bleeding risk showed some evidence that the intraoperative use of the CytoSorb® is a safe and effective method to reduce postoperative bleeding complications (29, 30). However, such promising data were mainly reported from one German institution, and additional evidence is required to establish and strongly recommend the use of such a hemoabsorber system to prevent bleeding and related complications in these patients.

In patients treated with vitamin K antagonists, the administration of 4-factor prothrombin complex concentrates (PCCs) is recommended (31). However, the optimal dosage and time point is unclear. According to a recent opinion of several European experts, PCC should be administered at a dose of 12.5–25 U/kg bodyweight (31). In clinical settings with increased thromboembolic risk, such as the perioperative period of cardiac surgery, PCC should be administered at the lower range of the suggested dose (31). Finally, the administration after heparin reversal with protamine rather than before CPB might be prudent according to the authors' opinion. In addition, the preoperative administration of vitamin K might be helpful for faster recovery of vitamin-K dependent coagulation factors in the postoperative period. No data on the preoperative administration of vitamin K in patients undergoing emergent surgery for ATAAD is available.

Similarly, the optimal treatment is not well defined in patients treated with DOACs, and it depends on the DOAC type. In patients treated with dabigatran, the preoperative use of idarucizumab might be considered (19). In contrast, the use of andexanet alfa in patients treated with direct FXa inhibitors is discouraged as andexanet alfa might interact with heparin and limit its anticoagulant activity during CPB (32). Recent clinical and *in vitro* data suggest that the use of the CytoSorb® hemoabsorber system during CPB might be able to eliminate directly the FXa inhibitors (29, 30, 33, 34), similar to ticagrelor removal. Again, more clinical data are warranted for stronger recommendations of the use of the CytoSorb® system in patients with recent intake of FXa inhibitors before emergent surgery for ATAAD. If massive bleeding occurs after cardiac surgery in patients with recent preoperative FXa inhibitors, the use of andexanet alfa might be considered. If urgent re-operation with the use of heparin is required early after administration of andexanet alfa, the alternative anticoagulants (e.g., direct intravenous thrombin inhibitor such as bivalirudin or argatroban) should be considered.

### 4.2. Ordering stable or labile blood products and coagulation factors concentrates

Postoperative bleeding is a common complication after emergent surgery in ATAAD patients. In addition, coagulopathy and bleeding might be present before and during surgery. However, it is unclear whether and which blood and coagulation products should be ordered before surgery. Several studies reported wide variations in the ordering of blood products with poor adoptions of guidelines and recommendations (35–37). A recent survey among European cardiac anesthesiologists confirmed significant differences in transfusion logistics and local transfusion practices at the different centers (35). Local institutional conditions, processes, and availabilities of coagulation products such as platelet concentrates, cryoprecipitate, or coagulation factor concentrates might be more important than international guidelines and evidence (35). Further, the presence and acuity of perioperative coagulopathy might influence ordering of blood products. Finally, specific patients' factors such as age, which has shown to increase the perioperative need of red blood (RBC) transfusions, or rare blood groups and the presence of RBC antibodies might relevantly influence the physician's decision to order specific blood products and coagulation factors in the individual patient.

## 5. Intra- and postoperative coagulation therapy measures

### 5.1. Transfusion strategies

The approach to transfusion in ATAAD may vary based on the institution, the specific patient's condition, the surgical team's preferences, and the latest evidence-based guidelines (3, 38). Two strategies are possible: Algorithm-based transfusion strategies involve predefined protocols or algorithms that guide clinicians in making transfusion decisions based on specific criteria. These criteria may include a combination of clinical parameters such as hemoglobin levels, hematocrit, patient symptoms, and specific triggers (standard laboratory values or viscoelastic testing results) for the administration of coagulation products outlined in the algorithm. The algorithm provides a step-by-step approach, which can help standardize transfusion decisions and reduce variability and is often accessible by all the disciplines. These algorithms are published widely an example of which is the Granducato algorithm (39). Evidence-based transfusion strategies are based on the best available scientific evidence from well-designed clinical trials and studies. Evidence-based approaches consider the results of research on the risks and benefits of transfusion in specific patient populations. The goal is to tailor transfusion decisions to individual patient needs while minimizing unnecessary transfusions. This approach requires ongoing review and incorporation of new research findings into clinical practice. However, given the nature of emergency thoracic aortic disease randomized clinical trials are difficult to undertake. The algorithm-based approach using viscoelastic testing has been associated with lower transfusion of allogeneic blood products and no difference in the use of coagulation factor concentrates (40). However, in emergent situations, a combination of both approaches is often used. Algorithms can be based on existing evidence while also being updated as new evidence emerges. This allows for a balance between standardized decision-making and incorporating the latest scientific knowledge.

### 5.2. Anticoagulation monitoring on cardiopulmonary bypass

Monitoring appropriate anticoagulation with unfractionated heparin during extracorporeal circulation is similar to other cardiac surgery procedures and is performed by determining the activated clotting time (ACT). According to the current guidelines an ACT above 480 s should be targeted during CPB with uncoated circuit equipment and cardiopulmonary suction (recommendation class IIa, evidence level C) (41). To achieve this value, a heparin dose of between 300 and 500 U/kg is usually required, depending on factors such as previous anticoagulant use or antithrombin III levels. It should be also noted that ACT is a general test of blood clotability and may be prolonged even without adequate doses of heparin. Especially in hypothermic patients or in patients who have received a large amounts of colloidal or crystalloid infusions (for example for the stabilization of hemodynamics in the preclinical phase), the ACT may be prolonged relevantly beyond the normal value (80–100 s) even without heparin. In accordance with the current guidelines, the authors consider the use of an individualized heparin management strategy with the Hemostasis Management System (Hepcon, Medtronic, Minneapolis, MN, USA) as a more sophisticated approach, especially for complex cardiac surgery (recommendation class IIa, evidence level B) (41). Hepcon has been available since the 1990 s and is used to determine the heparin dose to achieve an adequate plasma level of heparin (usually 3–4 U/ml) in the extracorporeal circuit. Repeated (ACT-independent) measurement of anticoagulation ensures a constant heparin level during surgery and allows precise protamine dose calculation (based on the current heparin level in the extracorporeal circuit and the patient). The latter is particularly important to avoid protamine overdose with associated bleeding complications and increased transfusion requirements (recommendation class IIa, evidence level B) (41, 42).

### 5.3. Coagulation testing and therapy after weaning from cardiopulmonary bypass

While monitoring of anticoagulation during CPB is limited to control and adjust ACT or heparin levels, determination of the individual coagulation potential is crucial before weaning patients after ATAAD repair from CPB. Surgical management of ATAAD requires hypothermic circulatory arrest and, accordingly, a prolonged (>3 h) extracorporeal perfusion time. The contact of the blood with the artificial surface of the extracorporeal circuit and the air in the surgical situs and in the venous reservoir induces a complex cascade of inflammatory processes, which, in addition to mechanical damage to the corpuscular elements of the blood and hypothermia, leads to consumption and dilution coagulopathy at the end of surgery. Typically, coagulation is severely deranged at the end of the perfusion period, with both impaired platelet function and a deficiency of clotting factors, particularly fibrinogen. In order to be able to objectively assess the coagulation and thus the need for blood products (including coagulation factor concentrates), it is advisable to perform coagulation testing as soon as possible after weaning from CPB or even already during CPB (Figure 2) (43).

Massive bleeding after weaning from CPB increases the duration of the operation and results in an increased requirement for transfusion of allogeneic blood products and increased mortality. Standard static tests from the laboratory such as platelet count, INR, aPTT, and Clauss fibrinogen are sometimes at odds with dynamic (point-of-care) viscoelastic tests (VET). The use of standard laboratory tests alone does not seem reasonable because they do not mirror the dynamic changes of the coagulation status. In this context, Clauss fibrinogen has lost its long preserved status as gold standard to assess fibrinogen contribution to clot. On the one hand, the determination of the plasmatic fibrinogen level according to the Clauss method takes a considerable time so that it is not suitable as guidance for fibrinogen therapy in ongoing bleeding situations as major cardiac surgery. On the other hand, the Clauss test is susceptible to variability and interference, especially with heparin and FDP (44).

In this context VETs have proved valid and useful in cardiac surgery (43). Rotational thromboelastometry (ROTEM), thromboelastgraphy (TEG) or techniques based on sonic estimation of elasticity via resonance (possibly complementary to platelet aggregometry) are able to assess all aspects of platelet (dys-)function, coagulation factor and fibrinogen deficiency allowing the clinician to make rapid interventions on the basis of the results.

A reasonable combination of VETs on CPB seems to be the performance of exTEM, fibTEM and inTEM assays in ROTEM or the kaolin-activated TEG, rapid TEG and functional fibrinogen assay in TEG (43). These functional tests cover the most common coagulation disorders (see above) and are suitable for ordering and preparing appropriate coagulation preparations such as fresh frozen plasma, PCC, fibrinogen concentrate and platelet concentrate (Figure 1). New test possibilities are offered by the Clotpro device (Haemonetics, Boston, Massachusetts, US), a more advanced variant of the ROTEM, with the possibility of screening for residual effect of FIIa and FXa inhibitors (if the patient had taken DOACs preoperatively) or performing the TPA test and thus determining the antifibrinolytic effect of TXA.

TXA is a synthetic lysine-analogue antifibrinolytic that functions by competitively inhibiting the transformation of plasminogen to plasmin, thus interrupting the dissolution and degradation of fibrin clots by plasmin. In the context of acute aortic dissection, maintaining the integrity of fibrin clots is paramount to prevent further propagation of the dissection or exsanguination. TXA displays significant potency in inhibiting plasmin-induced platelet activation, a mechanism that might be crucial in addressing hemostatic challenges in aortic dissection scenarios. At the cellular level, TXA's interaction with platelets is evidenced by its influence on the ADP-granule content, which could impact bleeding dynamics post-dissection. Moreover, TXA attenuates inflammatory cascades, suggesting a potential role in modulating systemic inflammatory responses associated with aortic injuries. The synergistic benefits of concurrent TXA and desmopressin administration may enhance platelet activation, thereby further reducing hemorrhagic events (45).

Incorporating this understanding of TXA, it becomes evident that its value isn't solely rooted in its molecular mechanism but also in its empirically demonstrated efficacy in reducing the need for transfusions and subsequent surgical interventions post-operatively, vital parameters in cardiac surgical care (46–49). Nevertheless, it's imperative to acknowledge the most significant reported side effect of TXA, namely convulsive seizures, which are a clearly dose-dependent (50, 51). The clinical application of TXA in acute aortic dissection demands a judicious approach, emphasizing evidence-based assessments and thorough risk-benefit analyses, all while considering specific dosing guidelines and potential side effects. For cardiac surgery, TXA dosing recommendations are moving more and more in the lower dose direction (52–54). One possible approach would be a singular bolus administration of only 20 mg/kg after anesthesia induction according to Zuffrey et al. (52). Another option is continuous administration of TXA, which may have advantages, especially in prolonged CPB runs, such as surgery for ATAAD. The TPA assay of the Clotpro device is a relatively new test to measure antifibrinolytic activity associated with TXA administration and could be used in prolonged surgery to adjust TXA administration as needed. However, it is currently unclear whether adjusting TXA dosing based on the results of the ClotPro assay could be beneficial in terms of optimized antifibrinolytic activity or reduced incidence of adverse effects in clinical practice.

### 5.4. Measures to reduce blood product usage

Surgical treatment of ATAAD necessarily involves surgical steps that expose blood to the pericardium or pleura. Treatment of this blood varies - traditionally it has been aspirated via cardiotomy suction then reinfused to the patient, but this is being cautioned against because of the highly activated coagulation and inflammation in this type of blood. Direct reinfusion of this blood has been shown to increase transfusion requirements and promote lung injury, neurologic damage, and cognitive decline (41, 55–57). If this blood is collected in a separate reservoir, it can either be reinfused, washed with a cell processing device, or discarded. We agree with the recommendations in the latest guidelines on cardiopulmonary bypass in adult cardiac surgery (41), which state that depending on patient factors such as preoperative hematocrit and body surface area, small volumes can be discarded and medium volumes should be processed. Due to the loss of plasmatic clotting factors and the limited processing speed of cellsaver devices, large volumes often need to be directly reinfused. Residual blood in the CPB circuit after weaning should be treated in a similar manner. In hypovolemia, direct reinfusion of this blood is recommended, whereas in normovolemic patients, it is recommended that this blood be processed by cell salvage or ultrafiltration to achieve a higher hematocrit and thus reduce the need for transfusion in the post-CPB phase.

### 5.5. Review of the success of the procoagulant measures

While it is standard to assess the adequacy of heparin reversal after protamine administration, evaluation of the efficacy of other measures to promote hemostasis is less standardized. This evaluation can be done either clinically by assessing bleeding tendency at the situs, or by coagulation testing in the laboratory. Even the specific guidelines for blood management in cardiac surgery do not address how and when to reassess bleeding tendency (3). We recommend regular clinical monitoring of bleeding tendency not only by the surgeon but also by the anesthesiologist. In addition, coagulation tests (traditional or viscoelastic tests) should be regularly repeated after procoagulant interventions to assess the effect and initiate the next therapeutic steps if necessary. This is particularly important if coagulopathy persists and thus clotting factors and platelets are being consumed. This reassessment should occur no later than arrival in the ICU. In cases of severe bleeding, we suggest shorter time intervals - e.g., 1 h after procoagulant measures - as relevant consumption coagulopathy should be expected.

### 5.6. Cooperation with surgeons and local measures to reduce bleeding

Because aortic dissection, surgery, and cardiopulmonary bypass all result in activation of coagulation pathways and consumption of clotting factors, perioperative coagulation disorders are very common in these patients. A common model of coagulation status helps avoid mutual blame. Bleeding is often a combination of surgical bleeding and impaired coagulation rather than due to a single cause (58).

Tissue sealants, adhesives, and hemostatic agents are substances administered directly into the wound to reduce bleeding and facilitate local clotting. Active agents utilize enzyme pathways involved in clotting, including fibrin sealants, topical thrombin, and externally administered antifibrinolytic drugs. In addition, passive adhesives such as collagen, gelatin, oxidized cellulose, and polysaccharide beads are used, as well as passive sealants such as cyanoacrylate, polyethylene glycol, and bovine albumin with glutaraldehyde. Some products even combine active and passive components. However, available studies are limited in scope and often carry a high risk of bias, in part due to the difficulty in blinding the surgical team. A recent systematic review concluded that the effectiveness of these measures remains uncertain (59). We suggest the use of these products as a second line when primary surgical hemostatic measures fail despite normal coagulation parameters. Timing is an important factor here: even these very localized measures take time to become effective, and the same is true for local compression with gauze. The art of successful collaboration is to coordinate the timing of blood product administration, the duration of local compression with gauze, and the exclusion of surgical or medical bleeding - all at a late hour! The experience of the team is the key to success here and cannot be compensated for by guidelines or specific measures.

### 5.7. Management of postoperative complications

Surgical treatment of acute type A aortic dissection is a procedure with high perioperative risk. Patients are particularly at risk for neurological complications, mainly due to malperfusion and ischemia of organs, and/or thromboembolism. The perioperative complications are associated with significant morbidity and mortality and may require further surgical or interventional treatment. Any evidence of organ dysfunction (clinically or by advanced monitoring methods such as NIRS, echo, or invasive blood pressure measurements) should prompt immediate diagnostic workup. Diagnostic workup should not be delayed until arrival in the ICU. To prevent thrombotic and thromboembolic complications, prophylactic anticoagulation with unfractionated heparin is usually initiated 6–12 h after surgery if the postoperative bleeding is within the normal range. As the increasing life expectancy of the population is likely to lead to an increase in the incidence of acute type A aortic dissection, the incidence of comorbidities will most likely increase as well. Especially in patients with indications for anticoagulation (e.g., atrial fibrillation, recent thromboembolic events) or dual platelet aggregation (e.g., recent implantation of a drug-eluting stent), postoperative anticoagulation measures and antithrombotic medication must be individualized.

Bleeding is a common complication after cardiac surgery. Hall et al. reported 34% coagulopathic bleeding in patients who required reoperation after cardiac surgery (60), and Choong et al. reported 22% coagulopathic bleeding (61). All anticoagulants must be discontinued and coagulation parameters rechecked because coagulation changes are sometimes very pronounced in the first postoperative hours. Delay in re-exploration has been shown to be related to postoperative mortality; therefore, coagulation testing should not delay surgical evaluation of the patient.

## 6. Summary

The perioperative hemostatic management of patients with type A aortic dissection requires unique expertise that goes beyond the scope of standard cardiac surgery cases. Diagnosis and therapy of coagulopathy is based on the results of (repeated) viscoelastic testing, taking into account the increased postoperative bleeding risk due to intraoperative application of moderate hypothermia and long extracorporeal circuilation time. Anticoagulant medication is administered after cardiac surgery in accordance with algorithms and guidelines in the best case, but also takes into account the clinical pattern in an interdisciplinary consensus. Postoperative neurological impairment requires immediate diagnosis, as it may be a consequence of organ ischemia, but also of thromboembolism.
